# Enhanced abscopal anti-tumor response via a triple combination of thermal ablation, IL-21, and PD-1 inhibition therapy

**DOI:** 10.1007/s00262-024-03718-1

**Published:** 2024-06-04

**Authors:** Shaoxian Wu, Hongwei Jiang, Zhang Fang, You Wu, Jing Jiao, Weiwei Fang, Yue Wu, Yanyan Lang, Ning Chen, Ziyang Zhong, Lujun Chen, Xiao Zheng, Binfeng Lu, Jingting Jiang

**Affiliations:** 1 Department of Tumor Biological Treatment, The Third Affiliated Hospital of Soochow University, Changzhou, 213003 Jiangsu China; 2 Jiangsu Engineering Research Center for Tumor Immunotherapy, The Third Affiliated Hospital of Soochow University, Changzhou, 213003 Jiangsu China; 3 Institute of Cell Therapy, The Third Affiliated Hospital of Soochow University, Changzhou, 213003 Jiangsu China; 4 Center for Discovery and Innovation, Hackensack Meridian Health, Nutley, NJ 07110 USA; 5 Shanghai Junshi Biosciences Co.,Ltd., Shanghai, 201206 China; 6 Anwita Biosciences Inc, San Carlos, CA 94070 USA

**Keywords:** Microwave ablation, Interleukin-21, Anti-tumor, Effector function

## Abstract

Despite the success of immune checkpoint inhibitors (ICIs) in treating solid tumors, lots of patients remain unresponsive to this therapy. Microwave ablation (MWA) stimulates systemic adaptive immunity against tumor cells by releasing tumor antigens. Additionally, IL-21 has demonstrated importance in stimulating T-cell effector function. The combination of these three therapies—MWA, IL-21, and anti-PD-1 monoclonal antibodies (mAbs)—has yet to be explored in the context of cancer treatment.

In this study, we explored the impact of thermal ablation on IL-21R expression in tumor-infiltrating lymphocytes (TILs). Subsequently, we assessed alterations in the tumor microenvironment (TME) and peripheral lymphoid organs. Additionally, we conducted a thorough examination of tumor-infiltrating CD45^+^ immune cells across various treatment groups using single-cell RNA sequencing (scRNA-seq). Moreover, we determined the potential anti-tumor effects of the triple combination involving MWA, IL-21, and anti-PD-1 mAbs.

Our findings revealed that MWA upregulated the expression of IL-21R on various immune cells in the untreated tumors. The combination of MWA with IL-21 exhibited a robust abscopal anti-tumor effect, enhancing the effector function of CD8^+^ T cells and facilitating dendritic cells' maturation and antigen presentation in the untreated tumor. Notably, the observed abscopal anti-tumor effect resulting from the combination is contingent upon T-cell recirculation, indicating the reliance of systemic adaptive immunity for this treatment regimen. Additionally, the combination of MWA, IL-21, and PD-1 mAbs demonstrated profound abscopal anti-tumor efficacy. Our findings provide support for further clinical investigation into a triple combination therapy involving MWA, IL-21, and ICIs for the treatment of metastatic cancer.

**Supplementary Information:**

The online version contains supplementary material available at 10.1007/s00262-024-03718-1.

## Introduction

Thermal ablation (TA), a local tumor treatment method, is widely used in colorectal cancer liver metastasis, hepatocellular carcinoma, and lung cancer [1–4]. TA, including MWA and radiofrequency ablation (RFA), induces local coagulation and necrosis, resulting in the release of tumor antigens, thereby activating the systematic anti-tumor adaptive immune response [4]. Preclinical studies have shown that TA can enhance tumor antigen-specific CD8^+^ T-cell function [5]. Combining TA with anti-PD-1, TIGIT, or LAG-3 mAbs can lead to stronger anti-tumor responses and prolonged survival in preclinical models [5–7]. However, TA alone does not completely prevent tumor growth. Incomplete ablation can even accelerate tumor progression [5, 8]. Therefore, understanding the immune mechanisms that limit the efficacy of TA is important.

Cytokines, primarily small protein molecules (typically less than 30 kDa in size), are synthesized by immune cells and serve as pivotal regulators in maintaining immune equilibrium while also influencing the development and progression of conditions such as tumors and autoimmune diseases [9]. IL-21 plays a pivotal role in anti-tumor immune responses due to its capacity to augment the cytotoxic activity of both CD8 ^+^ T cells and NK cells [10]. Under in vitro conditions, IL-21 suppresses Tregs expansion by inhibiting Foxp3 expression [11], and mice treated with IL-21 did not cause side effects such as in vivo toxicity of vascular leak syndrome even at high concentrations [12, 13]. The anti-tumor effects of IL-21 have been extensively studied in various preclinical mouse tumor models, combining IL-21 and immune checkpoint inhibitors exerts more effective anti-tumor effects [14–17]. In addition, recombinant IL-21 has been tested as an anti-tumor agent in various clinical trials, achieving good results in colorectal cancer and metastatic renal cell carcinoma [18, 19]. The outcomes of early clinical trials showed certain anti-tumor activities associated with IL-21.

Although therapies based on TA or IL-21 have exhibited promising advancements, they still encounter obstacles in achieving substantial tumor eradication. We are also motivated to determine whether these therapies can be combined with ICIs therapy. To that end, we started to examine whether TA treatment regulates the expression of IL-21R on tumor-infiltrating immune cells. We then investigated how combination treatment with MWA and IL-21 transformed the immune network within the TME. In addition, we studied whether systemic immune responses are required for the anti-tumor effects of the combination therapy. Finally, we addressed whether these therapies can be effectively combined with anti-PD-1 ICI therapy.

## Results

### MWA treatment led to an upregulation of IL-21R expression on T cells, while exerting limited impact on IL-2 family receptors

**Fig. 1:**
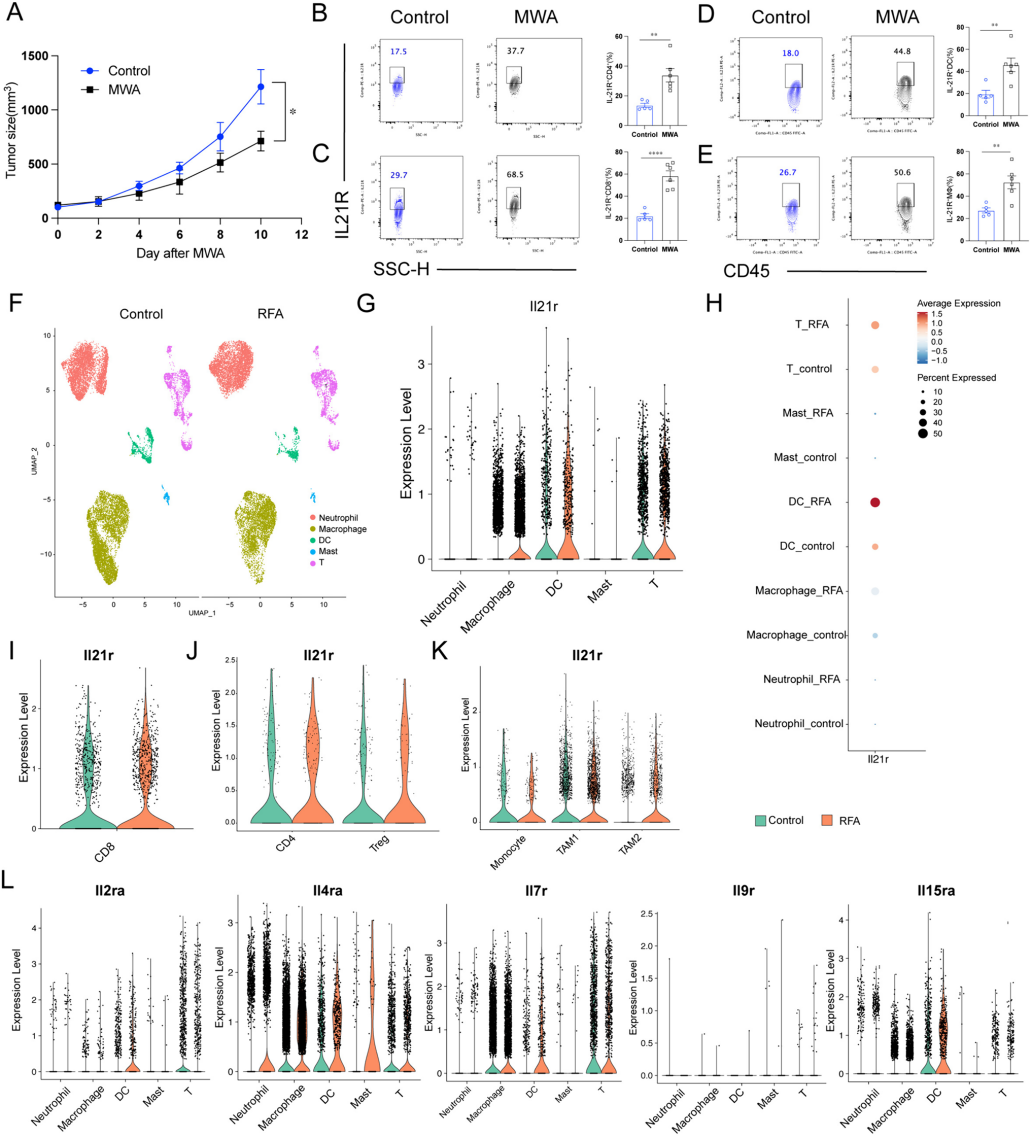
Ablation results in an upregulation of IL-21R expression in immune cells. **A**–**E** 1 × 10^6^ MC38 cells were subcutaneously inoculated into the bilateral flanks of C57BL/6J mice. At about 7 days, when the maximum diameter of the mouse tumor was about 7 mm, the tumor-bearing mice were randomly divided into different groups, and then, MWA (microwave ablation) was performed on one side of the tumor. The size of the tumor on the other side was measured every 2 days. **A** Tumor growth curves of tumor-bearing mice in the Control group and MWA group. **B**–**E** Representative flow plots and quantitative analysis of IL-21R expression on tumor-infiltrating CD4^+^ T cells, CD8^+^ T cells, dendritic cells (DCs), and macrophages. **F** Uniform manifold approximation and projection (UMAP) visualization of single-cell RNA sequencing of tumor-infiltrating immune cells from Control and post-RFA (radiofrequency ablation) pancreatic cancer Panco2 tumor-bearing mice. **G**, **H** Violin and Dot plots showing the distribution of IL-21R expression among neutrophils, macrophages, dendritic cells, mast cells, and T cells in both the Control and RFA groups. **I**–**K** Violin plots illustrate the expression distribution of IL-21R within CD8^+^ T cells, CD4^+^ T cells, Tregs, and myeloid cells in the Control and RFA. **L** Violin plots illustrate the distribution of IL-2 family receptors expression among neutrophils, macrophages, dendritic cells, mast cells, and T cells in both the Control and RFA groups.

To investigate the impact of MWA on the expression of IL-2 family receptors, we established the MC38 model of bilateral back tumors in wild-type C57BL/6J mice and performed flow cytometry analysis (sFig. 1A). Consistent with the previous results [5, 6], compared with the Control group, MWA inhibited tumor growth (Fig. 1A), upregulated IL-21R expression on CD8 ^+^ T cells, CD4^ +^ T cell, dendritic cells (DCs), and macrophages (Fig. 1B–E). Next, we analyzed published pancreatic cancer tumor-bearing mouse models [20] immune cell single-cell transcriptome sequencing data (Fig. 1F). RFA increased IL-21R expression in DCs and T cells (Fig. 1G and H). T cells were further divided into CD8 ^+^ T cells, CD4 ^+^ T cells, and Treg cells. We observed that RFA elevated the IL-21R expression in CD8 ^+^ T cells (Fig. 1I and J, sFig. 1B). We divided the myeloid cell population into monocytes, TAM1 and TAM2 (sFig. 1C), and the results showed that IL-21R expression was lower in myeloid subpopulations, and RFA did not affect its expression in these cells (Fig. 1K, sFig. 1D). Meanwhile, we also found that RFA had little effect on the expression of IL-2 family receptors among different immune cell populations (Fig. 1L and sFig. 1E). We discovered that RFA led to an elevation in the expression of IL-4RA while causing a reduction in the expression of IL-2RA, IL-7R, and IL-15RA on CD4 ^+^ T cells (sFig1F–G). This suggests that ablation-induced anti-tumor effects may require the participation of the IL-21/IL-21R signaling pathway.

### MWA combined with IL-21 synergistically inhibits tumor growth and alters the tumor microenvironment

**Fig. 2:**
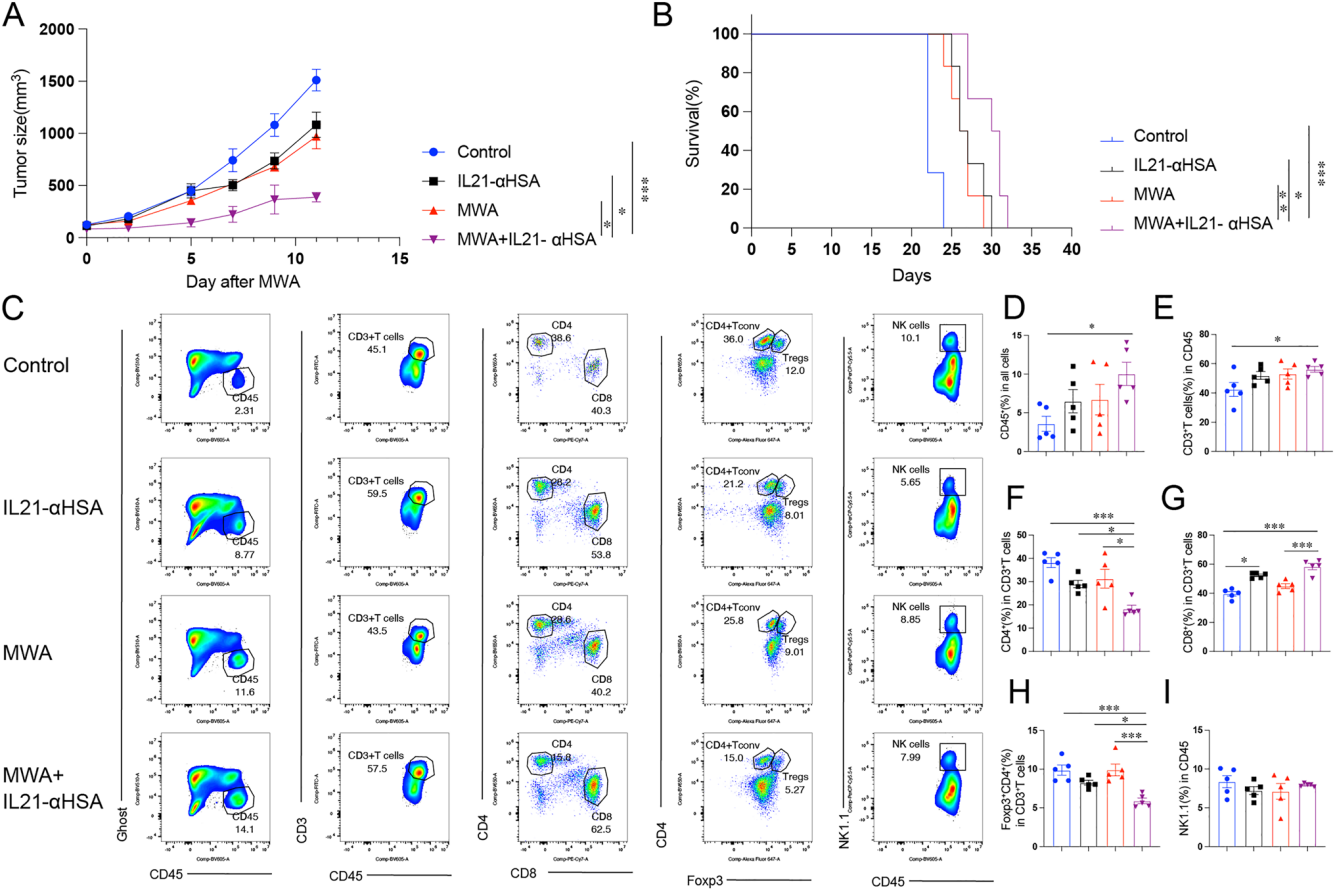
MWA combined with IL-21 significantly inhibited tumor growth and altered the tumor microenvironment. **A**–**B** MC38 tumor cells (1 × 10^6^) were subcutaneously inoculated into both sides of the back of C57BL/6J mice. Around 7 days, when the maximum diameter of the mouse tumor was about 7 mm, the tumor-bearing mice were randomly divided into different groups, and then, MWA was performed on the one side of the tumor. Twenty-four h later, the tumor-bearing mice were intraperitoneally injected with IL-21-αHSA (30 μg). Mice were humanly sacrificed when the maximum diameter of the tumor exceeded 20 mm or when the tumor was ruptured. Tumor volume (**A**) and Kaplan–Meier survival (**B**) were measured every other day. Data were presented as mean ± SEM, *n* = 5, *P < 0.05, **P < 0.01, ***P < 0.001, and ****P < 0.0001, two-way ANOVA test and the log-rank test were performed. **C**–**I** Tumor tissues were analyzed by flow cytometry 48 h after the two treatments. **C** Representative flow plots of tumor-infiltrating immune cell populations in different experimental groups. **D**–**I** Quantitative percentage of tumor-infiltrating CD45^+^ lymphocytes, CD3^+^, CD4^+^, CD8^+^, Foxp3^+^CD4^+^T cells, and NK cells. Data were presented as mean ± SEM, *n* = 5, *P < 0.05, **P < 0.01, ***P < 0.001, and ****P < 0.0001, one-way ANOVA test was performed.

Considering the pivotal role of IL-21/IL-21R in ablation therapy, we established colon cancer tumor-bearing mouse models individually. We used a previously published half-life extended IL-21 (IL-21-αHSA) [16]. The results showed that IL-21 enhanced the effect of MWA treatment in both MC38 model (Fig. 2A) and CT26 model (sFig. 2A). In addition, combined treatment prolonged the survival of mice (Fig. 2B). However, these favorable results were not replicated in the B16 model (sFig. 2B), a model much more resistant to immune therapy [21]. We further analyzed the TME immune cell population of MC38 tumor-bearing mice. MWA combined with IL-21 increased the infiltration of CD45^+^ immune cells and CD8^+^ T cells, and decreased the proportion of CD4^+^ T and CD4^+^ Foxp3^+^ T (Tregs, regulatory T cells) cells (Fig. 2C–I). Next, we also explored the changes of T cells in peripheral lymphoid organs mediated by MWA combined with IL-21. In the spleen, combination therapy significantly decreased the fraction of naïve CD62L^+^CD4^+^ and CD62L^+^CD8^+^ T cells. The fraction of effector CD44^+^CD4^+^ or CD44^+^CD8^+^ T cells was increased in the combination group (sFig. 3A and B–G). However, we did not find similar results in tumor-draining lymph nodes (TDLNs) (sFig. 3A and H–M). These data indicate that MWA combined with IL-21 increases immune cell infiltration and exerts a stronger anti-tumor effect.

### *The synergy between MWA and IL-21 inhibits tumor growth by bolstering the effector function of CD8*^+^*T cells*

**Fig. 3:**
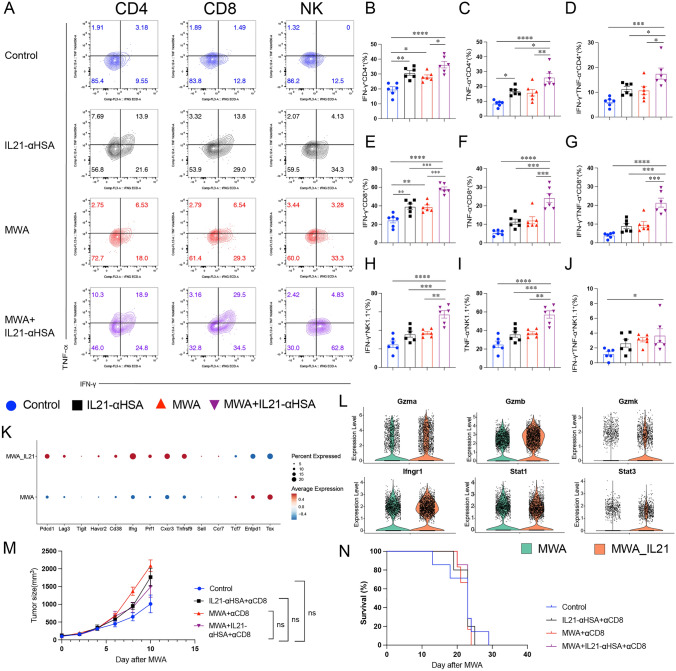
CD8^+^ T cells play a crucial role in augmenting the anti-tumor efficacy of IL-21-mediated microwave ablation. **A**–**L** The establishment of the MC38 tumor-bearing mouse model and treatment plan is the same as Fig. 2. Tumor tissues were analyzed by flow cytometry and single-cell transcriptome sequencing 48 h after the two treatments. **A** Representative flow plots showing IFN-γ and TNF-α staining in CD4^+^, CD8^+^ T cells, and NK cells. B-J. Quantitative percentage of IFN-γ and TNF-α expression in CD4^+^, CD8^+^ T cells, and NK cells. Data were presented as mean ± SEM, n = 6, *P < 0.05, **P < 0.01, ***P < 0.001, and ****P < 0.0001, one-way ANOVA test was performed. **K**–**L** Dot plot and violin showing the expression of the above molecules in CD8^+^ T cells within both MWA group and MWA combined IL-21 group. **M**–**N** CD8^+^ T cells were depleted by intraperitoneal injection of 200 μg anti-CD8 antibodies 1 day after MWA and once every 4 days. Tumor size (**M**) and Kaplan–Meier survival (**N**) curves were measured every other day. Data were presented as mean ± SEM, *n* = 5–7, *P < 0.05, **P < 0.01, ***P < 0.001, and ****P < 0.0001, two-way ANOVA test and the log-rank test were performed

To delve deeper into the mechanism underlying the augmentation of MWA anti-tumor efficacy by IL-21, we employed flow cytometry and scRNA-seq to study the tumor-infiltrating CD8^+^T cells. MWA combined with IL-21 synergistically increased the production of IFN-γ and TNF-α in CD4^+^, CD8^+^ T cells, and NK cells (Fig. 3A–J). Next, we performed unsupervised cluster analysis on the single-cell transcriptome sequencing data (sFig. 4A–D). We found that compared with the MWA group, MWA combined with IL-21 increased the expression of immune checkpoint molecules (PD-1, TIM-3, and LAG-3) and activation effector molecules (IFN-γ, PRF1, CXCR3, and TNFRSF9), while the expression of memory molecule TCF-1 (encode by *Tcf7*) was decreased (Fig. 3K). The violin plot showed that MWA combined with IL-21 increased the expression of GZMA, GZMB, GZMK, IFNGR1 and STAT3 (Fig. 3L). Relative to the MWA group, the combination of MWA with IL-21 sustained the expression of CD8^+^ T-cell activation effector genes and reduced the expression of naive/memory genes (sFig. 5 A–S). In addition, after the elimination of CD8^+^ T cells, we observed a diminished anti-tumor effect, irrespective of whether IL-21 alone, MWA, or the combined treatment was administered (Fig. 3M–N). These findings collectively suggest that CD8^+^ T cells play a pivotal role in the enhancement of MWA anti-tumor effects by IL-21. In summary, the therapeutic effects of both MWA and IL-21 rely on CD8^+^ T cells, and their combination further augments the anti-tumor potential of CD8^+^ T cells.

### scRNA-seq analysis reveals that the combined treatment of MWA and IL-21 leads to an increase in a CD8^+^ T-cell subset that expresses exhaustion markers and a higher expression of effector cytokines

**Fig. 4:**
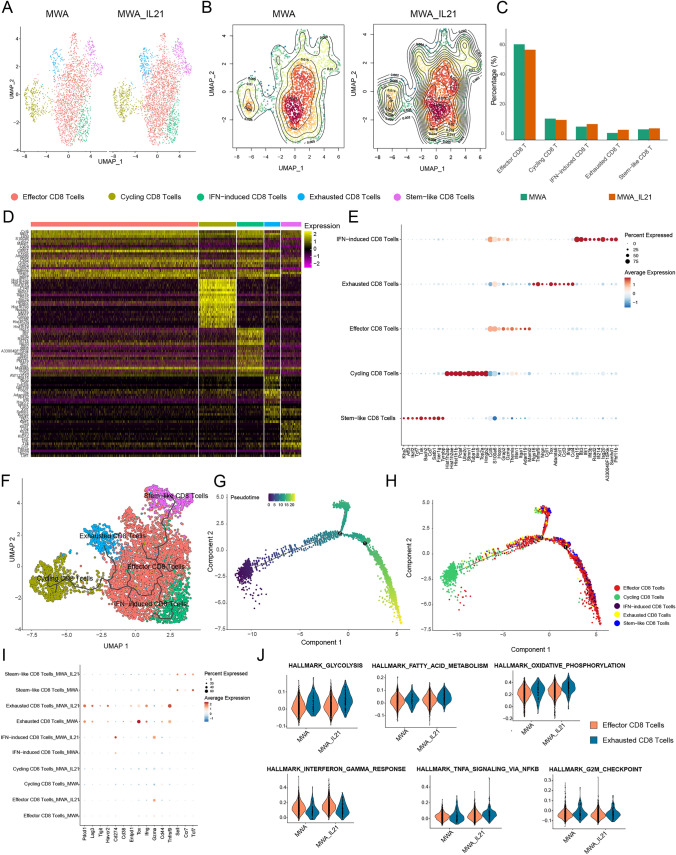
IL-21 reshapes the abundance and function of CD8^+^ T-cell subsets after MWA. **A**, **B** UMAP and Contour heatmap showing CD8^+^ T-cell subsets, including exhausted, cycling, stem-like, effector, and IFN-induced split by different treatment conditions. **C** Bar plots of CD8^+^ T-cell subsets in different treatment conditions. **D** Heatmap showing the expression distribution of top 20 genes in different CD8^+^ T-cell subsets. **E** Dot plot showing the expression distribution of top 10 genes in different CD8^+^ T-cell subsets. **F** Use the R package monocle3 (version 1.3.1) to infer the developmental trajectory of the CD8^+^ T-cell population and map it in the UMAP diagram. **G**, **H** Pseudotime plots of CD8^+^ T-cell population development were inferred and drawn using the R package monocle (version 2.26.0). **I** The dot plots show the expression distribution of the above genes in CD8^+^ T-cell subsets among different treatment groups. **J** The violin plots depict selected pathways regulated by effector CD8^+^ T cells and exhausted CD8^+^ T cells in the MWA group or the MWA combined with IL-21 group

Considering the pivotal role of CD8^+^ T cells in augmenting MWA's anti-tumor efficacy through IL-21, we categorized CD8^+^T cells into distinct subtypes, including effector, cycling, IFN-induced, exhausted, and stem-like (Fig. 4A–E) following the classification method outlined in our previously published articles [17]. We noted a pronounced presence of effector CD8^+^ T cells within the TME. Nevertheless, the combination of MWA and IL-21 led to a reduction in effector CD8^+^ T cells but an increase in the proportion of exhausted CD8^+^ T cells (Fig. 4C). Trajectory analysis indicated that CD8^+^ T cells followed a differentiation trajectory, progressing from stem-like to effector cell states and eventually developing into exhausted and cycling cell states (Fig. 4F–H). Despite the augmented proportion of exhausted CD8^+^ T cells, we also detected significant activation and the expression of effector molecules (Fig. 4I). We further analyzed the enriched pathways of effector CD8^+^ T cells and exhausted CD8^+^ T cells in different treatment groups. We found that pathways associated with fatty acid metabolism, oxidative phosphorylation responses were upregulated in the effector CD8^+^ T cells in the MWA plus IL-21 group. In addition, IFN-γ, TNF-α, glycolysis, fatty acid metabolism, and oxidative phosphorylation responses were upregulated in the exhausted CD8^+^ T cells in the MWA plus IL-21 group compared to the MWA group (Fig. 4J). In summary, these results indicate that the combination of MWA with IL-21 drives CD8^+^ T cells into hyperactivated cell populations.

### MWA combined with IL-21 enhances genes involved in antigen presentation and promotes DCs maturation

**Fig. 5:**
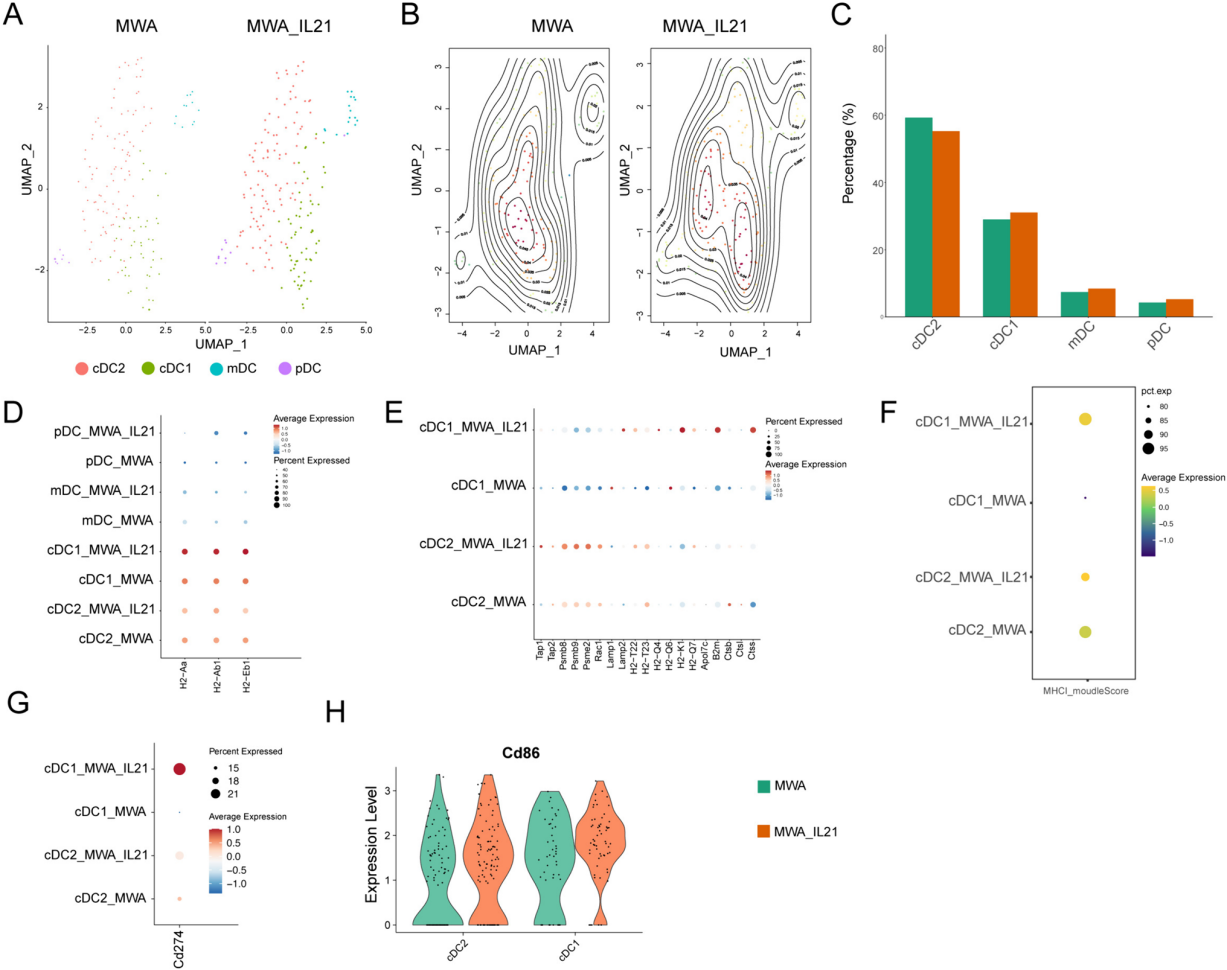
IL-21 augments antigen presentation and maturation of dendritic cells after MWA A-B. UMAP and Contour heatmap showing DCs subsets, including cDC1, cDC2, mDC, and pDC split by different treatment conditions. **C** Bar plots of DCs subsets in different treatment conditions. **D** The dot plots showing the expression distribution of the above genes in DCs subsets among different treatment groups. **E** The dot plots show the expression distribution of the above genes in cDC1 and cDC2 among different treatment groups. **F** Dot plot shows the average expression of MHC class I genes in cDC1 and cDC2. This function is implemented through the AddModuleScore function in the Seurat package in R. **G** The dot plots show the expression of *Cd274* in cDC1 and cDC2 among different treatment groups. **H** The violin plots show the expression of *Cd86* in cDC1 and cDC2 among different treatment groups

We also analyzed scRNA-seq data to explore the effect of MWA combined with IL-21 on dendritic cells within the TME (Fig. 5A and B). MWA combined with IL-21 decreased the proportion of type 2 dendritic cell (DC2) cells and increased the proportion of type 1 dendritic cell (DC1), mature/migratory DC (mDC), and plasmacytoid DC (pDC) (Fig. 5C). Subsequently, we also found that MWA combined with IL-21 increased the expression of MHC class I molecules in DC1 and enhanced their antigen presentation ability (Fig. 5D–F). Consistent with our speculation, MWA combined with IL-21 also increased the expression of costimulatory molecules CD86 and immunonegative molecules PD-L1 (*Cd274*) in DC1 (Fig. 5G and H). These findings indicate that IL-21 treatment can potentially augment the activation, maturation, and antigen presentation capabilities of DC within the tumor microenvironment following MWA.

### MWA combined with IL-21 alters macrophage function

Our previous data showed that MWA combined with anti-PD-1 mAbs increased the proportion of TAM1 cells and decreased the proportion of TAM2 cells [16]. Considering the substantial population of TAM within the TME, we conducted a scRNA-seq on monocytes, TAM1, and TAM2 (sFig. 6 A–B). We found that MWA combined with IL-21 increased the expression of tumor-associated macrophages (TAM) characteristic molecule CD14, and also upregulated the expression of immune-stimulating molecules such as TREM2, immunosuppressive molecules VEGFA, and PD-L1 (sFig. 6 C). The combined treatment of MWA with IL-21 additionally led to heightened expression of MHC molecules in TAM1 cells. As for monocytes, there was an increase in the expression of CD80, CD86, CXCL9, and CXCL10 (sFig. 6 D–F). For TAM2, MWA combined with IL-21 increased the expression of CCL2 and complement activation such as *C1qbp*, *C1qa*, and *C1qb* (sFig. 6 D–E).

### IL-21 enhances the cell–cell communication across all immune subsets after MWA

**Fig. 6:**
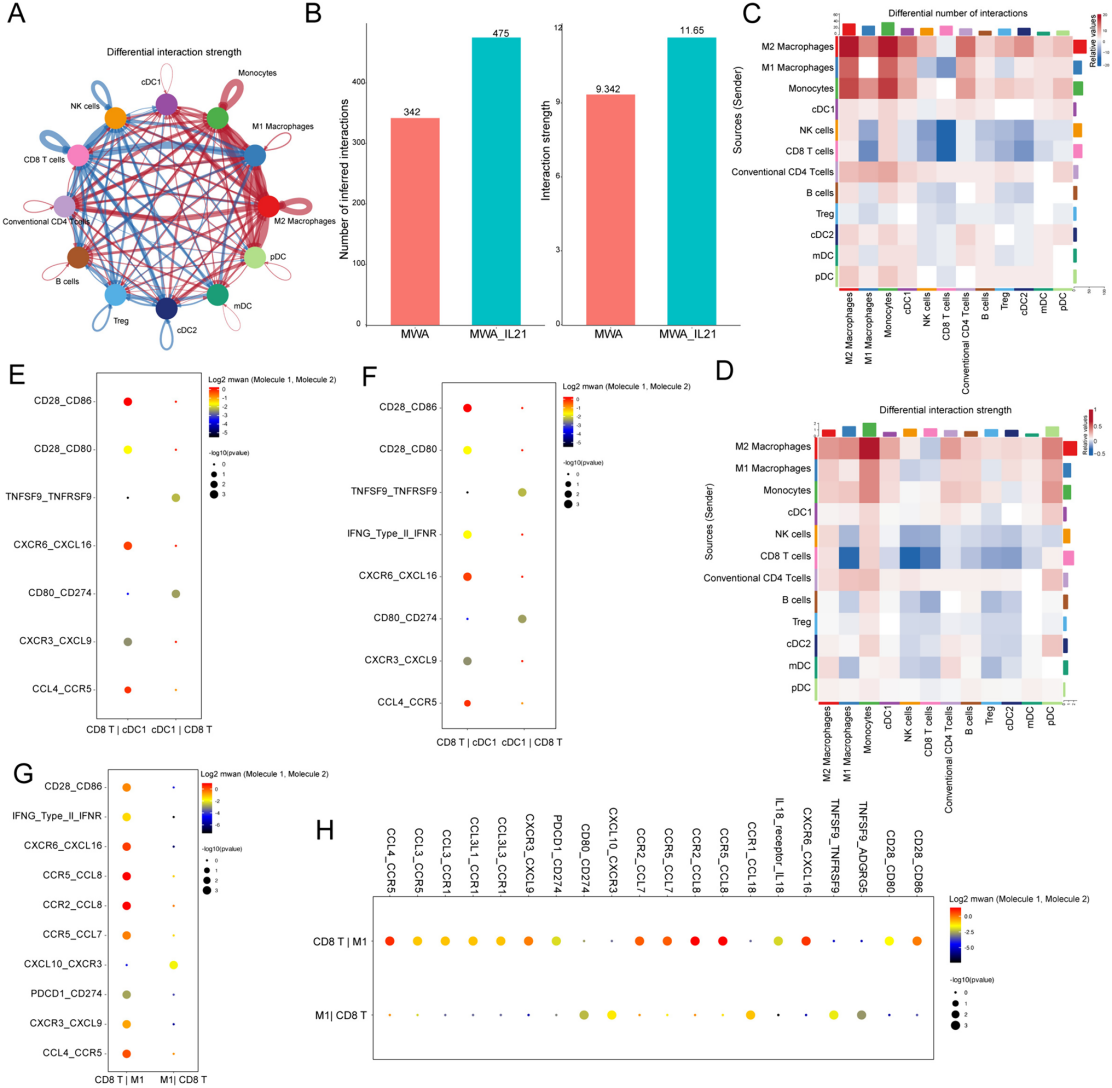
IL-21 enhances the cell–cell communication across all immune subsets after MWA. **A**–**F** The cell–cell communication among immune cell populations between the MWA group and MWA combined with IL-21 group. Blue represents a strong interaction among the immune cell populations of the MWA group, and red represents a strong interaction among the immune cell populations of the MWA combined with IL-21 group. **A**, **B** Use the CellChat package in R to infer and analyze the changes in the interactions of each cell population between the two data sets of MWA and MWA plus IL-21. **A**. Ring diagram. **B** Bar plot.** C-F**. Use CellPhoneDB to analyze the interaction changes of each cell population between the two data sets of MWA and MWA plus IL-21 based on the receptor, ligand, and interaction database, and use the heatmap_plot and dot_plot functions for visualization. **C**, **D** Heatmap showing the ligand–receptor interaction of CD8^+^T cells and DC1 between MWA (**E**) and MWA combined with IL-21 (**F**). Dot plots showing the ligand–receptor interaction of CD8^+^T cells and TAM1 between MWA (**G**) and MWA combined with IL-21 (**H**).

In order to further explore the interaction between cells, we use Cellchat and CellPhoneDB package to inferred intercellular communication networks. MWA combined with IL-21 can significantly enhance the number of inferred interactions and interaction strength between immune cells (Fig. 6A–C). The preceding findings indicated that the combination of MWA and IL-21 amplified CD8^+^ T cells effector function, augmented DCs maturation and antigen presentation. Consequently, we employed the "CellPhoneDB" tool to analyze the interaction dynamics among CD8^+^ T cells, DC1, and TAM1 ligand receptors (Fig. 6E–H). The combined treatment of MWA and IL-21 led to an upsurge in the expression of ligand–receptor interactions between CD8^+^ T cells and TAM1. These findings suggest that the synergy between MWA and IL-21 alters the interactions among immune cells, creating a more favorable environment for anti-tumor effects.

### IL-21 enhances the anti-tumor effect after MWA requires the T-cell recirculation

**Fig. 7:**
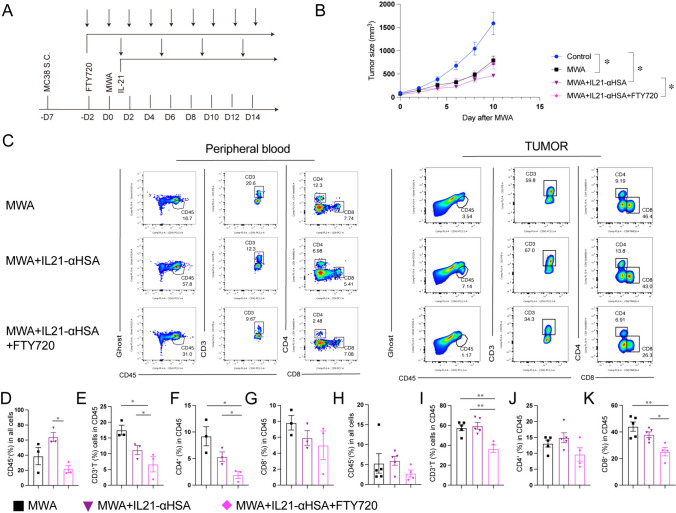
IL-21 enhances the anti-tumor effect after MWA requires the T-cell recirculation. **A**–**K** MC38 tumor cells (1 × 10^6^) were subcutaneously inoculated into both sides of the back of C57BL/6J mice, at about 7 days, when the maximum diameter of the mouse tumor was about 7 mm, the tumor-bearing mice were randomly divided into different groups, and then, MWA was performed on the one side of the tumor. Twenty-four h later, the tumor-bearing mice were intraperitoneally injected with IL-21-αHSA (30 μg). Thereafter, treat once every 4 days, for a total of four times. FTY720 was injected intraperitoneally during the first 2 days of MWA and every other day thereafter. Tumor size was measured every other day (**A**, **B**). Data were presented as mean ± SEM, *n* = 5, ^*^P < 0.05, **P < 0.01, ***P < 0.001, and ****P < 0.0001, two-way ANOVA test was performed. On the 21th day, peripheral blood and tumor tissues of different treatment groups were collected for flow cytometry analysis (**C**–**K**). **C** Representative flow plots of peripheral blood and tumor-infiltrating immune cell populations in different experimental groups. Quantitative percentage of peripheral blood (**D**–**G**) and tumor-infiltrating (**H**–**K**) CD45^+^ lymphocytes, CD3^+^, CD4^+^, and CD8^+^ T cells. Data were presented as mean ± SEM, *n* = 3–5, *P < 0.05, **P < 0.01, ***P < 0.001, and ****P < 0.0001, one-way ANOVA test was performed

MWA was performed on the one side, and tumor growth on the contralateral side was assessed to determine the resulting abscopal effect. Further investigation is required to explore whether tumor-specific T cells migrate through the lymph nodes into the contralateral TME. We injected mice with FTY720, an S1P receptor inhibitor that blocks T cells trafficking [17], 2 days before MWA. Blocking lymphocyte efflux counteracted the anti-tumor effect of MWA combined with IL-21 (Fig. 7A and B). Next, we performed flow cytometry analysis of immune cells in PBMC, spleen, TDLN, and tumor. FTY720 led to a decrease in the proportion of CD45^+^ immune cells and T cells in the peripheral blood and tumors of mice in the MWA/IL-21/FTY720 (Fig. 7C–K). The same results were found in TDLN; however, the change in the proportion of immune cell populations in the spleen was not very obvious (sFig. 7A–I). FTY720 did not affect the proportion of MDSC and DCs population after ablation, but compared with MWA combined IL-21 group, MWA/IL-21/FTY720 decreased the proportion of TAM1 and increased the proportion of TAM2 (sFig. 7J–P). These results indicate that FTY720 hinders T cells from exiting the lymph node and migrating to the TME through circulation, consequently diminishing the anti-tumor effect of the combined MWA and IL-21 therapy.

### The combination of IL-21 with anti-PD-1 mAbs was more effective in augmenting the anti-tumor effector of MWA

**Fig. 8:**
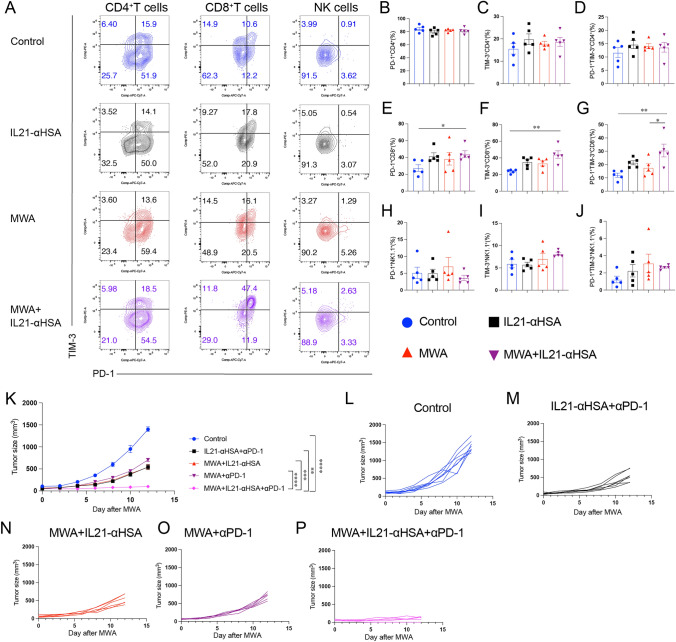
The combination of IL-21 with anti-PD-1 mAbs was more effective in augmenting the anti-tumor effector of MWA. **A-J** The establishment of MC38 tumor-bearing mouse model and treatment plan are the same as Fig. 2. Tumor tissues were analyzed by flow cytometry 48 h after the two treatments. **A** Representative flow plots showing PD-1 and TIM-3 staining in CD8^+^ T and CD4^+^ T and NK cells. **B**–**J** Quantitative percentage of PD-1 and TIM-3 expression in CD8^+^ T, CD4^+^ T, and NK cells. Data were presented as mean ± SEM, n = 5–7, *P < 0.05, **P < 0.01, ***P < 0.001, and ****P < 0.0001, one-way ANOVA test was performed. **K**–**P** MC38 tumor cells (1 × 10^6^) were subcutaneously inoculated into both sides of the back of C57BL/6J mice, at about 7 days, when the maximum diameter of the mouse tumor was about 7 mm, the tumor-bearing mice were randomly divided into groups, and then, microwave ablation was performed on the one side of the tumor. Twenty-four h later, the tumor-bearing mice were intraperitoneally injected with IL-21-αHSA (30 μg) or αPD-1 (200 μg). Thereafter, treat once every 4 days, for a total of four times. Tumor size was measured every other day (**K**). **L**–**P**. Individual mouse tumor growth curves. Data were presented as mean ± SEM, *n* = 5, *P < 0.05, **P < 0.01, ***P < 0.001, and ****P < 0.0001, two-way ANOVA test was performed

Exhausted T cells and NK cells will highly express immune checkpoint molecules, which can impact the efficacy of combination therapy [22]. Next, we checked the expression of PD-1 and TIM-3 by flow cytometry. Compared with the Control group, we found that MWA combined with IL-21 significantly increased the expression of PD-1 and TIM-3 on the surface of CD8^+^T cells, and significantly increased the proportion of PD-1^+^TIM-3^+^CD8^+^T cells (Fig. 8A–J). This is also consistent with the previous results of single-cell transcriptome sequencing (Fig. 3K). This shows that MWA combined with IL-21 leads to the production of hyperactivated/exhausted cell populations. Therefore, we intraperitoneally injected IL-21 and anti-PD-1 mAbs into mice 24 h after MWA. Our simultaneous administration of IL-21 and anti-PD-1 mAbs after MWA can effectively inhibit tumor growth in mice (Fig. 8K–P). Our study shows that IL-21 can be combined with checkpoint inhibitors to improve the anti-tumor effect of MWA.

## Discussion

As an economical treatment approach with minimal impact on surrounding tissues [23, 24], ablation is widely used in the treatment of malignant tumors such as kidney cancer, liver cancer, and lung cancer [25–28]. Shi et al. demonstrated that RFA enhanced T-cell effector function in the contralateral tumor TME, but it was unable to induce sustained effects in the later stages of tumor-bearing mice [5]. This suggests that there are also other negative signals regulating tumor growth in the later stages of ablation. IL-21 plays an important role in the anti-tumor immune response and has been tested as an anti-tumor drug in various preclinical and clinical models [16–19, 29]. In this study, we have found that combining MWA with IL-21-αHSA can enhance the anti-tumor effect and prolong the survival of tumor-bearing mice. The mechanism by which combination therapy produces a synergistic anti-tumor effect includes increasing the infiltration of CD45 + immune cells and CD8 + T cells. We also found that the combined anti-tumor effect of IL-21 and MWA is dependent on circulation of immune cells. Ultimately, the combination of MWA with IL-21 and anti-PD-1 mAbs further amplified the anti-tumor effect.

IL-21 is produced by CXCL13 ^+^ CD4 ^+^ T cells in human colon cancer, liver cancer, and non-small cell lung cancer. This cell population highly expresses molecules such as PD-1 and IFN-γ, and actively responds to PD-L1 blockade treatment [17, 30]. These results suggest that the expression of IL-21 in tumor tissues promotes anti-tumor immune responses. Our results indicate that ablation promotes high expression of IL-21R by tumor-infiltrating immune cells, including T cells, macrophages, and DCs. Although studies have combined ablation with ICIs in mouse colon cancer models to synergistically inhibit tumor growth, in the late stages of tumors, immune cells are exhausted, and the tumors cannot be completely eradicated [6, 7]. Our results show that MWA combined with IL-21 inhibits tumor growth and prolongs the survival of mice. Combination therapy increased the effector function of TILs. The anti-tumor effect produced by combination therapy requires the participation of CD8 ^+^ T cells. In addition, studies investigating the combined ablation of additional IL-2 family members have also been undertaken, injection of IL-7 and IL-15 after RFA effectively inhibits tumor growth in a mouse breast cancer model [31]. These results indicate that the ablation-induced anti-tumor effect of CD8 ^+^ T cells requires IL-21/IL-21R signaling pathway. In the future, histological staining will be performed on patients with ablated recurrent colorectal cancer liver metastases to determine the expression distribution of IL-21R, which provides a basis for MWA combined with IL-21 treatment.

MWA combined with IL-21 leads to an increase in the proportion of DC1 and a decrease in the proportion of DC2. Simultaneously, the expression of molecules associated with antigen presentation on the surface of DC1 cells increases, the expression of the activating and costimulatory molecules CD86 and PD-L1 was also elevated. Recent research results also indicate that the combination of IL-21 and anti-PD-1 mAbs produces similar effects [16, 17]. The anti-tumor mechanism produced by ablation includes the uptake of antigens by DCs and their migration into the TDLN to activate T cells, and the spread of tumor antigen-specific T cells to the contralateral tumor through the TDLN [32]. Marieke et al. demonstrated that both TDLN deletion and FTY720 experiments abolished the anti-tumor effects of PD-1 [33]. Nagasaki et al. demonstrated that PD-1 blockade therapy promoted the expansion of tumor antigen-specific CD8 ^+^ T cells from tumor-draining lymph nodes [34]. Our results found that the use of FTY720 before ablation abolished the synergistic anti-tumor effect of MWA combined with IL-21, and immune cells in peripheral blood and TME significantly decreased. These results indicate that tumor-reactive T cells, activated by antigens released from ablated tumors, must migrate to contralateral tumors to exert their anti-tumor activities.

A study on liver cancer patients with percutaneous thermal ablation found that the peripheral blood of patients with early recurrence contained a high proportion of PD-1^+^CD4^+^ T cells and TIM-3^+^CD8^+^ T cells [35]. A retrospective study included a total of 127 patients treated with anti-PD-1 plus RFA or RFA alone for recurrent HCC. The results showed that the 1-year recurrence-free survival rates of the anti-PD-1 plus RFA group and RFA group were 32.5% and 10.0%, respectively. RFA combined with PD-1 monoclonal antibody produced better results than RFA alone [36]. IL-21 was fused with an anti-PD-1 (PD-1Ab21) to target tumor-reactive T cells and exhibited potent anti-tumor effects [15]. In addition, an Erbitux-based IL-21 tumor-targeting fusion protein (Erb-IL-21) can extend the half-life of IL-21, effectively expand cytotoxic T lymphocytes, and improve its anti-tumor efficacy [37]. Recently, Zhou et al. constructed Anti-Claudin18.2-IL-21 fusion protein, which showed stronger anti-tumor effect and better safety [38]. The activation of CD8 ^+^ T cells caused by MWA combined with IL-21 is also accompanied by the increase in immune checkpoint molecules PD-1 and TIM-3. MWA has been previously studied in combination with PD-1 or TIGIT or LAG-3 mAbs [5–7]. IL-21 combined with PD-1 or CTLA-4 monoclonal antibodies can slow down tumor growth and increase CD8 ^+^ T-cell proliferation and infiltration [14]. Our findings demonstrate that the combination of MWA with IL-21 and anti-PD-1 mAbs is highly effective in suppressing tumor growth. These data show that IL-21 is important in the anti-tumor effect of MWA combined with anti-PD-1 mAbs. Our data also provide a novel strategy to clinically prevent recurrence in patients undergoing ablation for colorectal cancer liver metastases.

A significant limitation of our study is that we utilized transplant colon cancer models, with tumor cells being inoculated intradermally rather than employing orthotopic models. In addition, although the PD-1 blockade is highly effective and has become the standard of care for patients with deficient mismatch repair (dMMR) metastatic colorectal cancers (mCRCs), the same regimen has shown poor response rates (RRs) in patients with proficient mismatch repair (pMMR)/Microsatellite Stable (MSS) mCRCs. However, a recent clinical trial shows that a significant proportion (about 30%) of early-stage pMMR/MSS CRCs have pathological responses (PRs) to a combination of ipilimumab and nivolumab in the neoadjuvant setting [39, 40]. Furthermore, recent phase 2 clinical trials exploring combination therapies, such as anti-CTLA-4 plus anti-PD-1 or anti-PD1 plus the HDAC inhibitors chidamide alongside bevacizumab, have produced promising efficacy with manageable toxicity in pMMR/MSS mCRCs [41, 42]. Together, these results support the potential effectiveness of immunotherapy as a viable treatment choice for pMMR/MSS mCRCs in the near future. Thus, we are hopeful that our regimens can be integrated into future clinical trials. For example, IL-21 and thermal ablation could potentially be combined with anti-CTLA-4 and anti-PD-1 therapies for CRC with liver metastasis.

## Materials and methods

### Mouse

C57BL/6J and BALB/c mice (6–8 weeks old) were purchased from Changzhou Cavens Model Animal Co., Ltd. and maintained in a specific pathogen-free (SPF) animal facility in the third-floor breeding room of Building 1 of Changzhou Cavens Model Animal Co., Ltd. (No. 211, Tanjiatou, Wujin District, Changzhou, China, 213,104). All mouse experiments were approved by the Ethics Committee of the Third Affiliated Hospital of Soochow University.

### Cell culture and tumor model

MC38, CT26, and B16 cell lines were originally originated from the Chinese Academy of Sciences, Shanghai Institutes for Biological Sciences [5], and were passaged and stored in the No. 2 liquid nitrogen tank of Department of Tumor Biological Treatment of the Third Affiliated Hospital of Soochow University. MC38 cells were cultured in DMEM medium containing 10% fetal bovine serum (FBS) and 1% penicillin–streptomycin (P.S). CT26 cells and B16 cells were cultured in RPMI-1640 medium containing 10% fetal bovine serum (FBS) and 1% penicillin–streptomycin (P.S). 1 × 10^6^ MC38 cells were inoculated subcutaneously into the bilateral flanks of C57BL/6J mice. 0.5 × 10^6^ CT26 or B16 cells were subcutaneously inoculated into BALB/C and C57BL/6J mice on bilateral flanks, respectively. For how to use MWA, please refer to previously published articles [6]. Briefly, MWA was performed on the one side of the tumor when its maximum diameter reached approximately 7 mm. MWA is performed using ablation electrodes inserted percutaneously into the center of the tumor. Treatment was performed at 70 °C and 10 W for 2.5–5 min. IL-21-αHSA (30 μg) or αPD-1 (200 μg) treatment was performed 24 h after MWA. The drug was injected intraperitoneally into the mice every 4 days. To deplete CD8^+^ T cells, mice were intraperitoneally injected with 200 mg of anti-CD8 antibody (clone 2.43, BioXcell, USA) starting 1 day after MWA and then every 4 days. FTY720 was injected intraperitoneally during the first 2 days of MWA and every other day thereafter. Tumor size was measured every other day, and the tumor volume was calculated as L × W^2^/2.

### Reagents and antibodies

IL-21-αHSA was kindly provided by Shanghai Junshi Biosciences Co., Ltd. (Shanghai, China), PD-1mAb (clone J43), CD8mAb (Clone:2.43, BioXcell, USA), and hamster IgG were purchased from Bioxcell company (Catalog No. BE0091). FTY720 (catalog no. 402615-91-2) was obtained from Cayman Chemical. Ghost Dye Violet 510 was purchased from Cell Signaling Technology (catalog no. 59863S). Flow cytometry, CD45 (clone 30-F11), CD3 (clone 17A2), CD4 (clone GK1.5), CD8 (clone 53–6.7), NK1.1 (clone PK136), Foxp3 (clone MF-14), PD-1 (clone 29F.1A12), TIM-3 (clone RMT3-23), CD62L (clone MEL-14), CD44 (clone IM7), IFN-γ (clone XMG1.2), TNF-α (clone MP6-XT22), GzmB (clone QA16A02), CD11b (clone M1/70), Ly6C (clone HK1.4), Ly6G (clone 1A8), F4/80 (clone BM8), CD11C (clone N418), CD103 (clone M290), and CD206 (clone MR6F3) were purchased from Biolegend, eBioscience, and BD Bioscience.

### Processing of tissues and flow cytometry

For the processing and staining procedures of mouse lymph nodes, spleens, and tumors, please refer to our previous articles [16]. Briefly, the lymph nodes and spleen were placed in 2 mL of serum-free 1640 medium and squeezed with the frosted surface of a glass slide to prepare a single-cell suspension. Spleen cells require ACK to lyse red blood cells. Tumor tissues were placed in six-well plates containing serum-free 1640 medium, cut into small pieces, and digested with 0.25 mg/mL Liberase TL (Roche) and 0.33 mg/mL DNase 1 (Sigma) at 37 °C for 30 min. Then stop with 10% FBS 1640 to prepare a single-cell suspension for staining. Flow cytometry analysis was performed with DX FLE (Beckman) and analyzed using Flowjo software.

### scRNA-seq

The prepared tumor single-cell suspension was incubated with CD45 (TILs) Microbead Mouse Kit (Catalog No. 130-110-618, Miltenyi Biotec, Lerden, the Netherlands), CD45^+^TILs were then isolated according to the magnetic-activated cell sorting (MACS) protocol. Then follow the above staining steps to perform Ghsot dye and CD45-Percp staining. Finally, sorting was performed by FACS Aria II (BD Biosciences). The sorted cells were tested for cell viability through AOPI, and a T100^™^ Thermal Cycler (Bio-Rad) was used to perform single-cell transcriptome generation at 53 °C to generate cDNA templates. According to the manufacturer, the scRNA-seq library was generated using a Single Cell 3′ Library. Libraries were sequenced using an Illumina Novaseq6000 sequencer to a sequencing depth of at least 50,000 reads per cell using a paired-end 150-bp (PE150) read strategy (performed by iomics, Beijing).

### scRNA-seq data processing

scRNA-seq data obtained from 10× Genomics were matched to the mm10 mouse reference genome and quantified using Cell Ranger software. Cell Ranger survival data use the R package Seurat (version 4.3.0.1) to create Seurat objects. Further quality control of cells was performed using DoubletFinder software, including total UMI counts, number of genes detected, and proportion of mitochondrial gene counts per cell. Cells with UMI counts exceeding 5000 and mitochondrial gene counts exceeding 10% were screened out and then subjected to dimensionality reduction and unsupervised clustering analysis. Different cluster groups were defined based on the characteristic genes of mouse immune cell groups in our previously published article.

### Differential gene expression analysis

Differentially expressed genes between different groups were analyzed by EdgeR package (version 3.40.2). The original data obtained from the Seurat object are normalized using TMM (trimmed mean of *M* values), and the estimateDisp function is used to estimate the dispersion of gene expression values. Use the dot plot function of the Seurat package to visualize the selected DEGs.

### Trajectory analysis of T cells

The Seurat object of CD8^+^T cells was converted into the corresponding cds object through the monocle3 package (version 1.3.1) in R, and the developmental trajectory was constructed through the learn_graph function. Visualization is performed through the plot_cells function. Single-cell developmental trajectories were constructed in CD8 T cells using the Monocle R package (version 2.26.0). The screening criteria for genes are expression in at least 10 cells.

### Statistical analysis

GraphPad Prism 8.0 software was used for graphing and statistical analysis. Data are expressed as mean ± SEM. Two-tailed unpaired Student's t-test was used between two groups, and one-way analysis of variance was used to quantitatively compare data between multiple groups. Tumor growth curves were compared between different groups using two-way ANOVA. The survival of mice was analyzed using the Kaplan–Meier method and calculated using the log-rank test. Significance levels were defined as ns (not significant, *P* > 0.05), ^*^P < 0.05, ^**^P < 0.01, ^***^P < 0.001, and ^****^P < 0.0001.

### Supplementary Information

Below is the link to the electronic supplementary material.

Supplementary Figure 1. Expression of IL-2 family receptors in control and RFA tumor-infiltrating immune cell subsets.** A** Flow Cytometry Gating Strategy Diagram. CD4^+^ T cells: CD45^+^CD3^+^CD4^+^, CD8^+^ T cells: CD45^+^CD3^+^CD8^+^, Tregs: CD45^+^CD3^+^CD4^+^Foxp3^+^, CD4^+^Tconv: CD45^+^CD3^+^CD4^+^Foxp3^-^, NK cells: CD45^+^CD3^−^NK1.1^+^, Ly6C^+^MDSC: CD45^+^CD11b^+^Ly6C^+^, Ly6G^+^MDSC: CD45^+^CD11b^+^Ly6G^+^, dendritic cells (DCs): CD45^+^Ly6G^−^MHCII^+^CD11C^+^, TAM1 macrophages: CD45^+^Ly6G^−^MHCII^+^F4/80^+^CD206^+^, and TAM2 macrophages: CD45^+^Ly6G^−^MHCII^+^F4/80^+^CD206^+^.** B** Dot plots showing the distribution of IL-21R expression among CD8^+^ T cells in both the Control and RFA groups.** C** UMAP visualization of single-cell transcriptome sequencing of myeloid cells from Control and post-RFA pancreatic cancer Panco2 tumor-bearing mice.** D** Dot plots illustrate the distribution of IL-21R expression among CD8^+^T and myeloid cells in both the Control and RFA groups.** E**–**G** Dot plots and violin plots illustrate the distribution of IL-2 family receptors expression among T-cell subsets in both the Control and RFA groups. (TIF 15385 KB)

Supplementary Figure 2. MWA combined IL-21 significantly inhibited tumor growth in multiple tumor model** A**,** B** CT26 (0.5×10^6^) and B16 (0.5×10^6^) tumor cells were separately subcutaneously inoculated into the bilateral flanks of BALB/c and C57BL/6J mice, when the maximum diameter of the mouse tumor was about 7 mm, the tumor-bearing mice were randomly divided into groups, and then, microwave ablation was performed on the one side of the tumor. Twenty-four h later, the tumor-bearing mice were intraperitoneally injected with IL-21-αHSA (30 μg), thereafter, treat once every 4 days, for a total of four times. Tumor volume (**A **and** B**) were measured every other day. Data were presented as mean ± SEM,* n* = 5-8, * P<0.05, ** P<0.01, *** P<0.001, and **** P<0.0001, two-way ANOVA test was performed. (TIF 3971 KB)

Supplementary Figure 3. Combination of MWA with IL-21 amplifies activation of peripheral immune cells.** A**–**M** The experimental design scheme is shown in Fig. 2. Peripheral tissues were analyzed by flow cytometry 48 h after the two treatments.** A. **Representative flow cytometry dot plots showing CD44 and CD62L staining of CD4^+^ T cells and CD8^+^ T cells in spleen and TDLN of different treatment groups.** B**–**M** The quantification result graph shows the expression ratio of CD44 and CD62L in the spleen (**B-G**) and TDLN (**H**–**M**) of different treatment groups in CD4^+^T cells and CD8^+^T cells. (TIF 11148 KB)

Supplementary Figure 4. Visualization of single-cell transcriptome sequencing of tumor-infiltrating CD45^+^ immune cells.** A** UMAP visualization of single-cell transcriptome sequencing of tumor-infiltrating immune cells from Control and post-MWA MC38 tumor-bearing mice.** B** Heatmap showing the expression distribution of top 20 genes in tumor-infiltrating immune cells subsets.** C** Dot plot showing the expression distribution of top 10 genes in tumor-infiltrating immune cells subsets.** D** UMAP visualization of single-cell transcriptome sequencing of T cells subsets from Control and post-MWA MC38 tumor-bearing mice. (TIF 18391 KB)

Supplementary Figure 5. Relative to the MWA group, the combination of MWA with IL-21 sustained the expression of CD8^+^ T-cell activation effector genes and reduced the expression of naive/memory genes.** A**–**S**. Pseudotime gene expression curves in CD8^+^ T cells in different treatment groups. (TIF 15244 KB)

Supplementary Figure 6. MWA combined with IL-21 treatment alters macrophage function.** A**,** B** UMAP and Contour heatmap visualization of single-cell transcriptome sequencing of monocytes, TAM1, and TAM2 from Control and post-MWA MC38 tumor-bearing mice.** C** The violin plot showing the expression of* Trem2, Cd14, Vegfa, and Cd274* in myeloid cell subsets within both MWA group and MWA combined with IL-21 group.** D**–**F** The dot plots display the expression patterns of the above genes within myeloid cell subsets across various treatment groups. (TIF 14720 KB)

Supplementary Figure 7. The enhanced anti-tumor effect of MWA combined with IL-21 requires the participation of TDLN.** A**–**P** The experimental scheme is the same as Fig. 7.** A** Representative flow plots of spleen and TDLN immune cell populations in different experimental groups. Quantitative percentage of spleen (**B**–**E**) and TDLN (**F**–**I**) CD45^+^ lymphocytes, CD3^+^, CD4^+^, and CD8^+^ T cells. J. Representative flow plots of tumor infiltration immune cell populations in different experimental groups.** K**–**P** Quantitative percentage of tumor infiltration MDSC, TAM1, TAM2, and DCs. (TIF 19045 KB)

## Data Availability

All raw data presented in this study are available from the corresponding author on reasonable request.
