# Supplementary figures and images for: Enhanced abscopal anti-tumor response via a triple combination of thermal ablation, IL-21, and PD-1 inhibition therapy
Source: Cancer Immunol Immunother. 2024 Jun 4;73(8):138. doi: 10.1007/s00262-024-03718-1 (PMC11150342; doi:10.1007/s00262-024-03718-1)

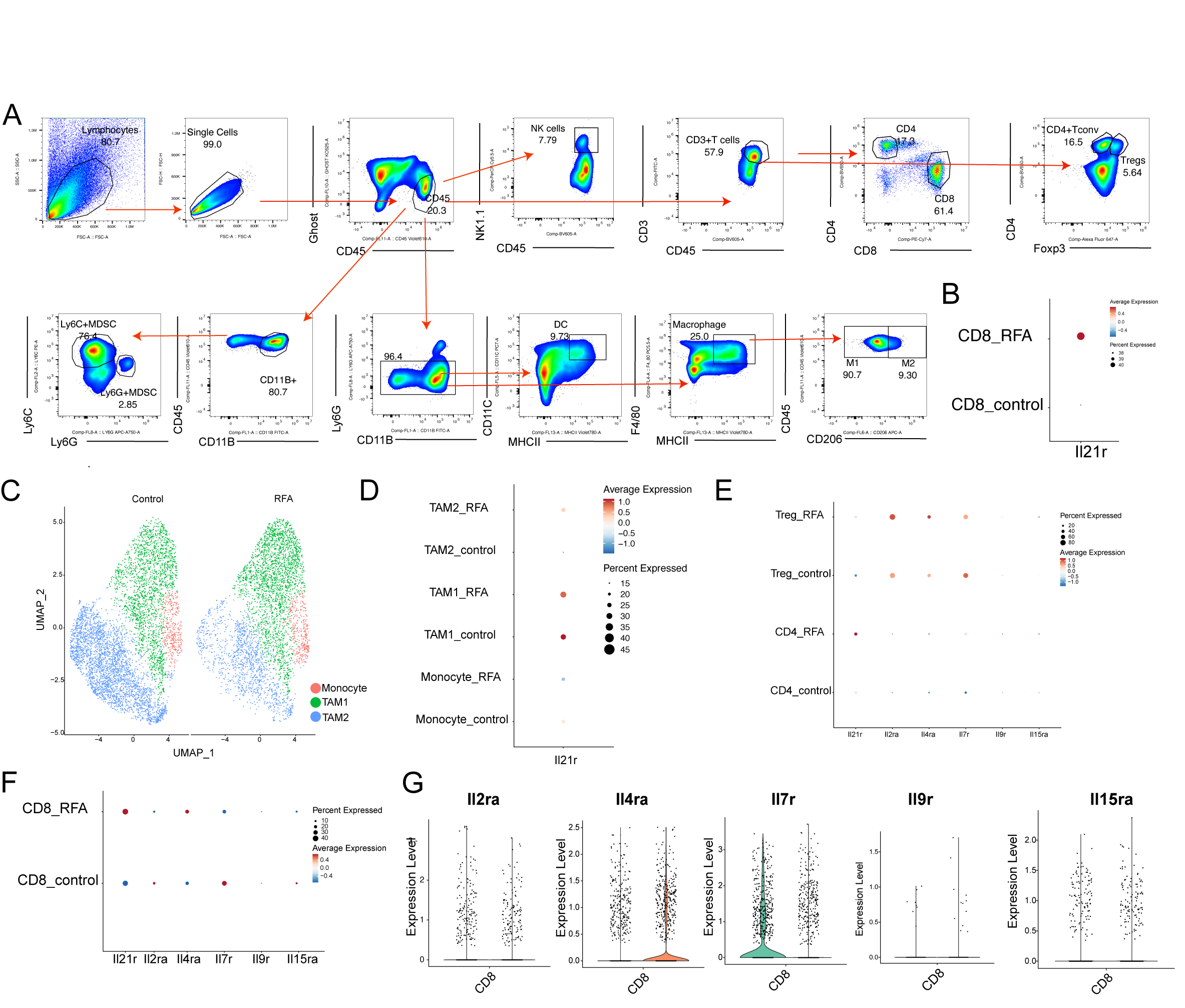

Supplement: Supplementary file 1 — Supplementary Figure 1. Expression of IL-2 family receptors in control and RFA tumor-infiltrating immune cell subsets. A Flow Cytometry Gating Strategy Diagram. CD4+ T cells: CD45+CD3+CD4+, CD8+ T cells: CD45+CD3+CD8+, Tregs: CD45+CD3+CD4+Foxp3+, CD4+Tconv: CD45+CD3+CD4+Foxp3-, NK cells: CD45+CD3−NK1.1+, Ly6C+MDSC: CD45+CD11b+Ly6C+, Ly6G+MDSC: CD45+CD11b+Ly6G+, dendritic cells (DCs): CD45+Ly6G−MHCII+CD11C+, TAM1 macrophages: CD45+Ly6G−MHCII+F4/80+CD206+, and TAM2 macrophages: CD45+Ly6G−MHCII+F4/80+CD206+. B Dot plots showing the distribution of IL-21R expression among CD8+ T cells in both the Control and RFA groups. C UMAP visualization of single-cell transcriptome sequencing of myeloid cells from Control and post-RFA pancreatic cancer Panco2 tumor-bearing mice. D Dot plots illustrate the distribution of IL-21R expression among CD8+T and myeloid cells in both the Control and RFA groups. E–G Dot plots and violin plots illustrate the distribution of IL-2 family receptors expression among T-cell subsets in both the Control and RFA groups. (TIF 15385 KB) [file 262_2024_3718_MOESM1_ESM.tif]

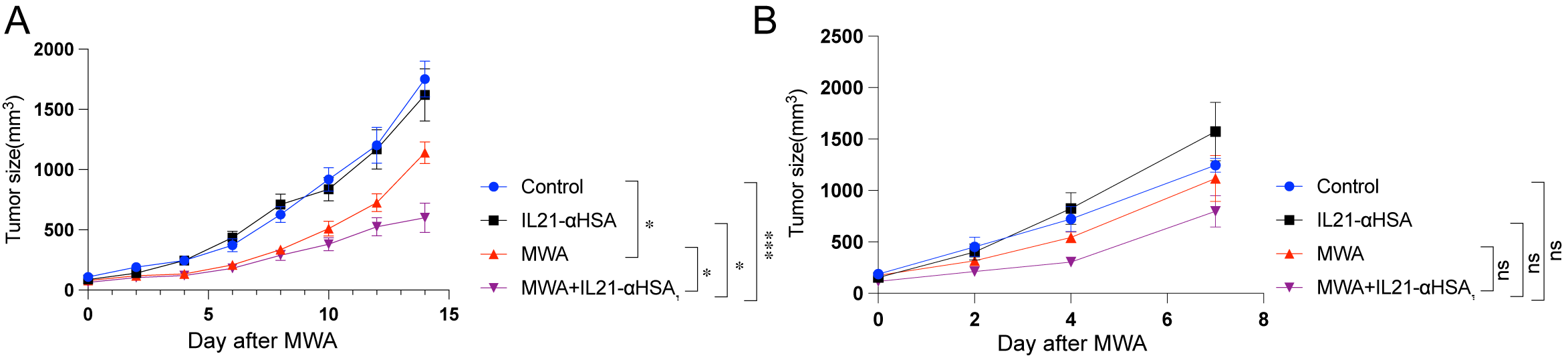

Supplement: Supplementary file 2 — Supplementary Figure 2. MWA combined IL-21 significantly inhibited tumor growth in multiple tumor model A, B CT26 (0.5×10^6^) and B16 (0.5×10^6^) tumor cells were separately subcutaneously inoculated into the bilateral flanks of BALB/c and C57BL/6J mice, when the maximum diameter of the mouse tumor was about 7 mm, the tumor-bearing mice were randomly divided into groups, and then, microwave ablation was performed on the one side of the tumor. Twenty-four h later, the tumor-bearing mice were intraperitoneally injected with IL-21-αHSA (30 μg), thereafter, treat once every 4 days, for a total of four times. Tumor volume (A and B) were measured every other day. Data were presented as mean ± SEM, n = 5-8, * P<0.05, ** P<0.01, *** P<0.001, and **** P<0.0001, two-way ANOVA test was performed. (TIF 3971 KB) [file 262_2024_3718_MOESM2_ESM.tif]

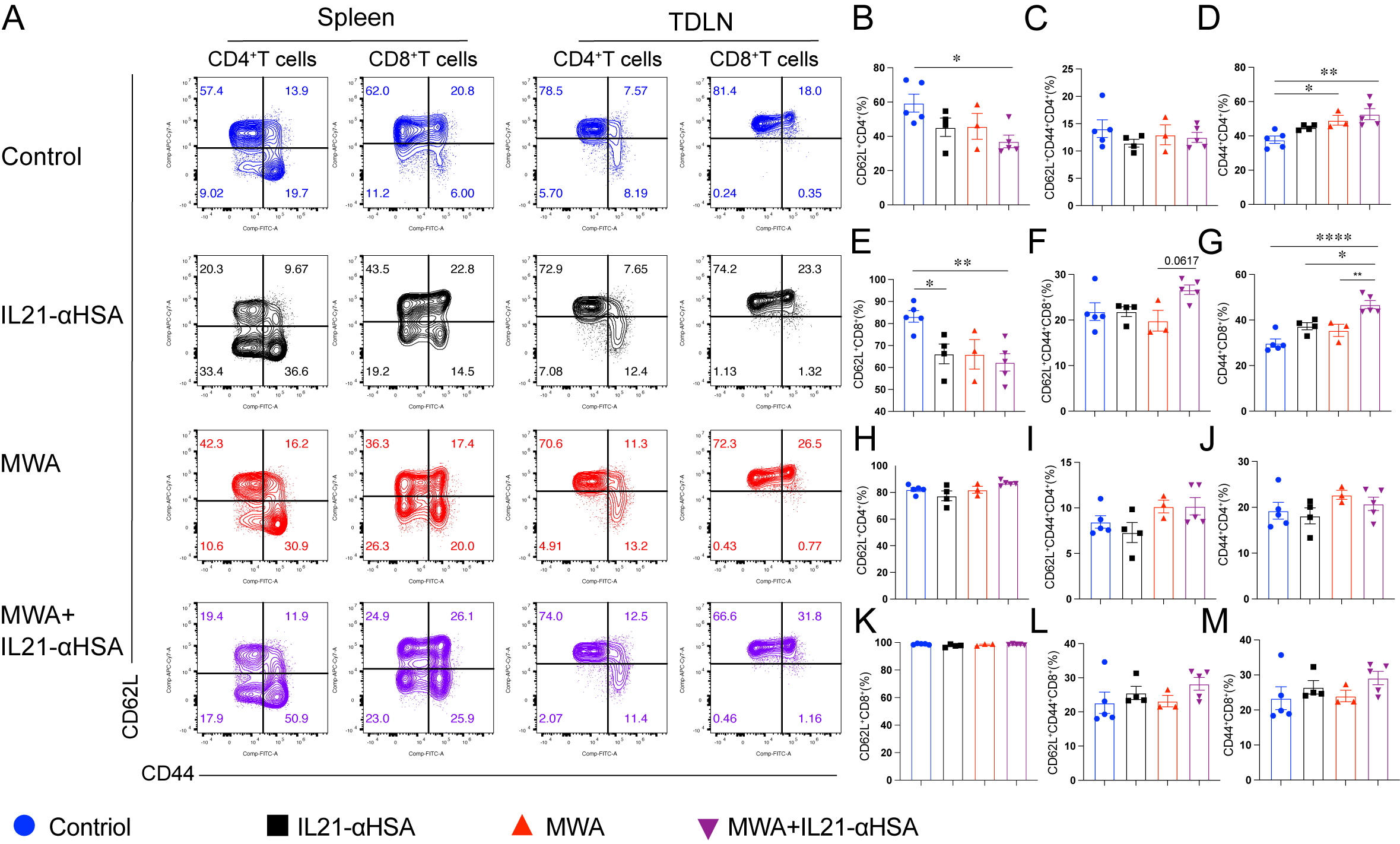

Supplement: Supplementary file 3 — Supplementary Figure 3. Combination of MWA with IL-21 amplifies activation of peripheral immune cells. A–M The experimental design scheme is shown in Fig. 2. Peripheral tissues were analyzed by flow cytometry 48 h after the two treatments. A. Representative flow cytometry dot plots showing CD44 and CD62L staining of CD4+ T cells and CD8+ T cells in spleen and TDLN of different treatment groups. B–M The quantification result graph shows the expression ratio of CD44 and CD62L in the spleen (B-G) and TDLN (H–M) of different treatment groups in CD4+T cells and CD8+T cells. (TIF 11148 KB) [file 262_2024_3718_MOESM3_ESM.tif]

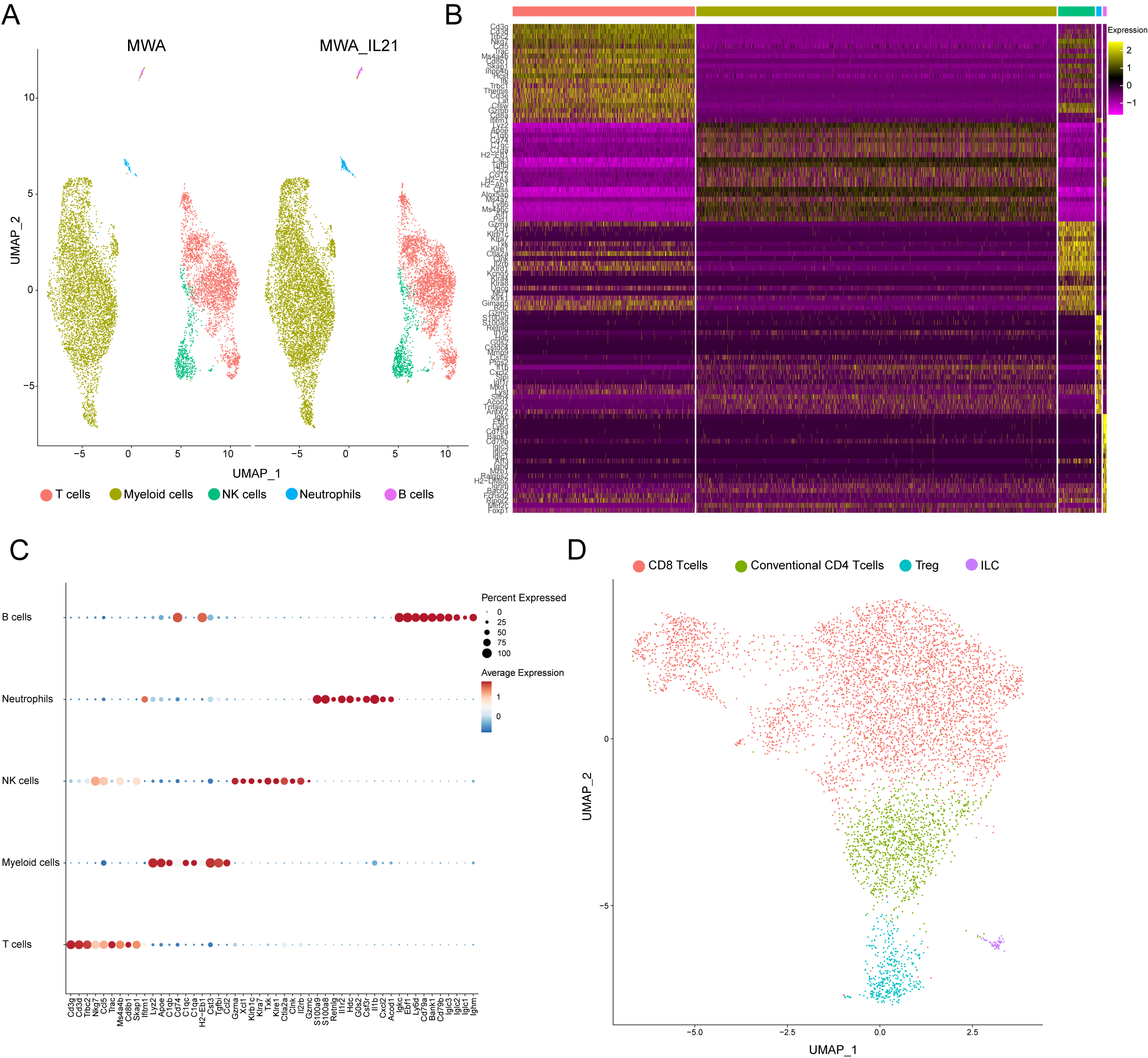

Supplement: Supplementary file 4 — Supplementary Figure 4. Visualization of single-cell transcriptome sequencing of tumor-infiltrating CD45+ immune cells. A UMAP visualization of single-cell transcriptome sequencing of tumor-infiltrating immune cells from Control and post-MWA MC38 tumor-bearing mice. B Heatmap showing the expression distribution of top 20 genes in tumor-infiltrating immune cells subsets. C Dot plot showing the expression distribution of top 10 genes in tumor-infiltrating immune cells subsets. D UMAP visualization of single-cell transcriptome sequencing of T cells subsets from Control and post-MWA MC38 tumor-bearing mice. (TIF 18391 KB) [file 262_2024_3718_MOESM4_ESM.tif]

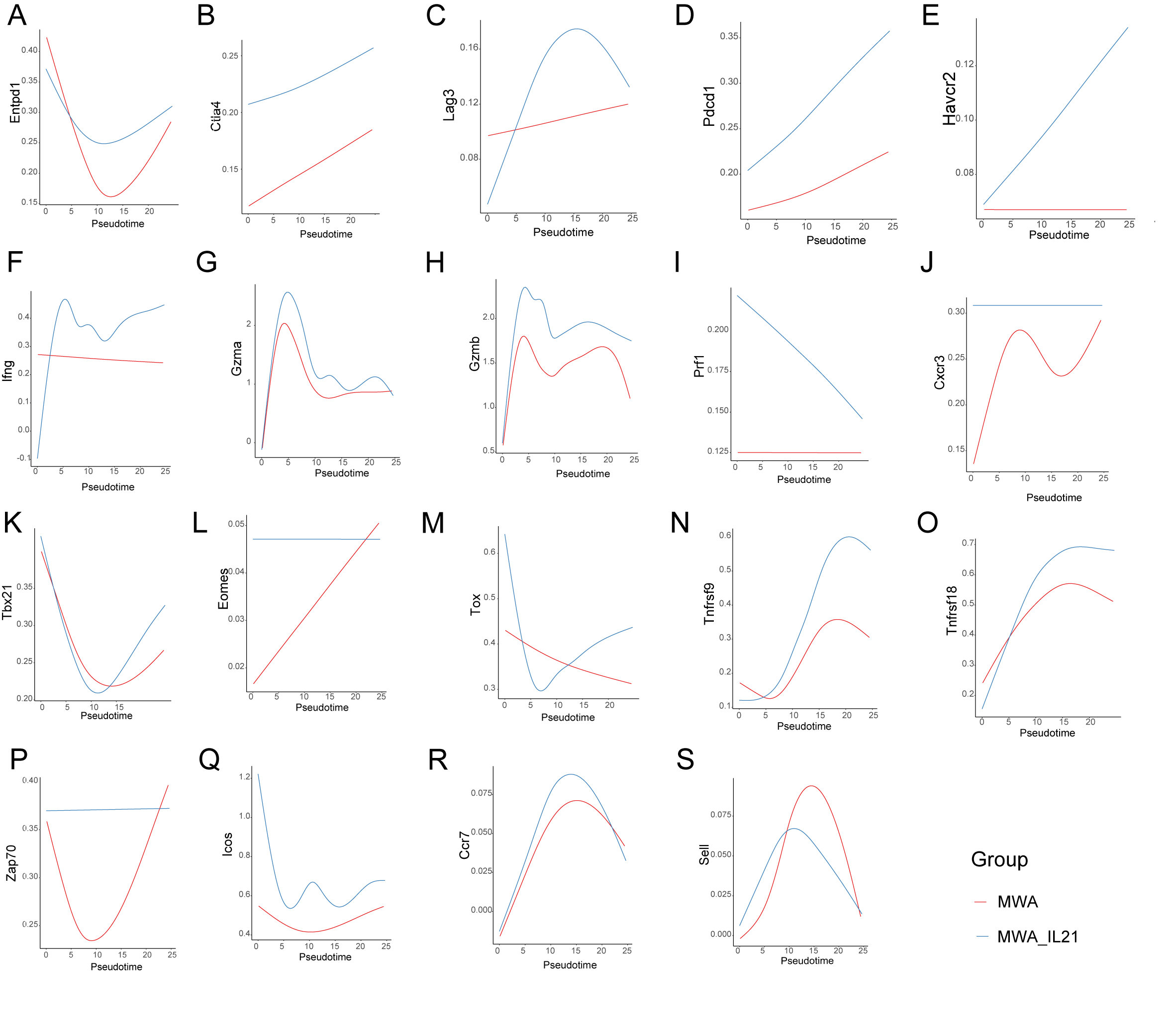

Supplement: Supplementary file 5 — Supplementary Figure 5. Relative to the MWA group, the combination of MWA with IL-21 sustained the expression of CD8+ T-cell activation effector genes and reduced the expression of naive/memory genes. A–S. Pseudotime gene expression curves in CD8+ T cells in different treatment groups. (TIF 15244 KB) [file 262_2024_3718_MOESM5_ESM.tif]

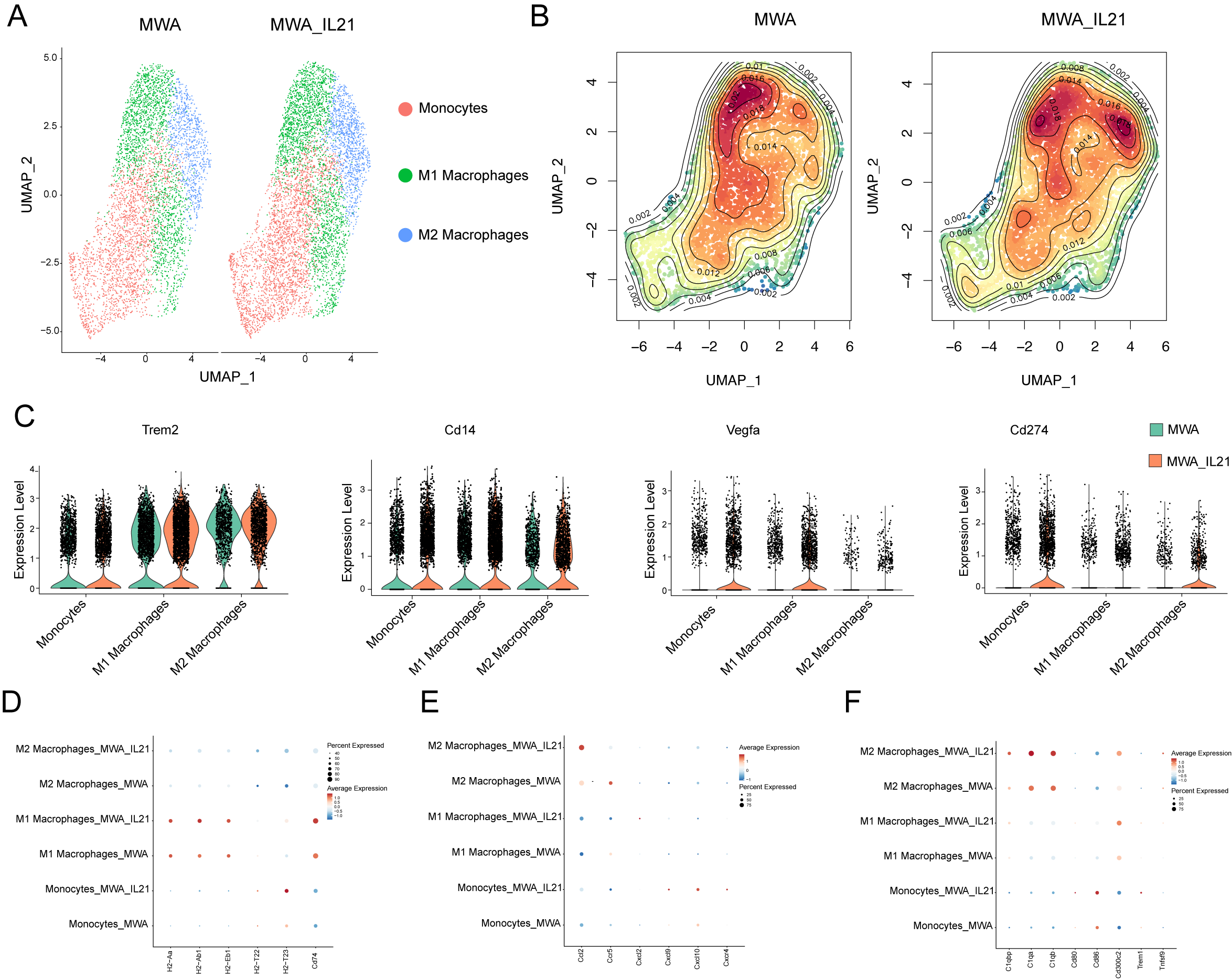

Supplement: Supplementary file 6 — Supplementary Figure 6. MWA combined with IL-21 treatment alters macrophage function. A, B UMAP and Contour heatmap visualization of single-cell transcriptome sequencing of monocytes, TAM1, and TAM2 from Control and post-MWA MC38 tumor-bearing mice. C The violin plot showing the expression of Trem2, Cd14, Vegfa, and Cd274 in myeloid cell subsets within both MWA group and MWA combined with IL-21 group. D–F The dot plots display the expression patterns of the above genes within myeloid cell subsets across various treatment groups. (TIF 14720 KB) [file 262_2024_3718_MOESM6_ESM.tif]

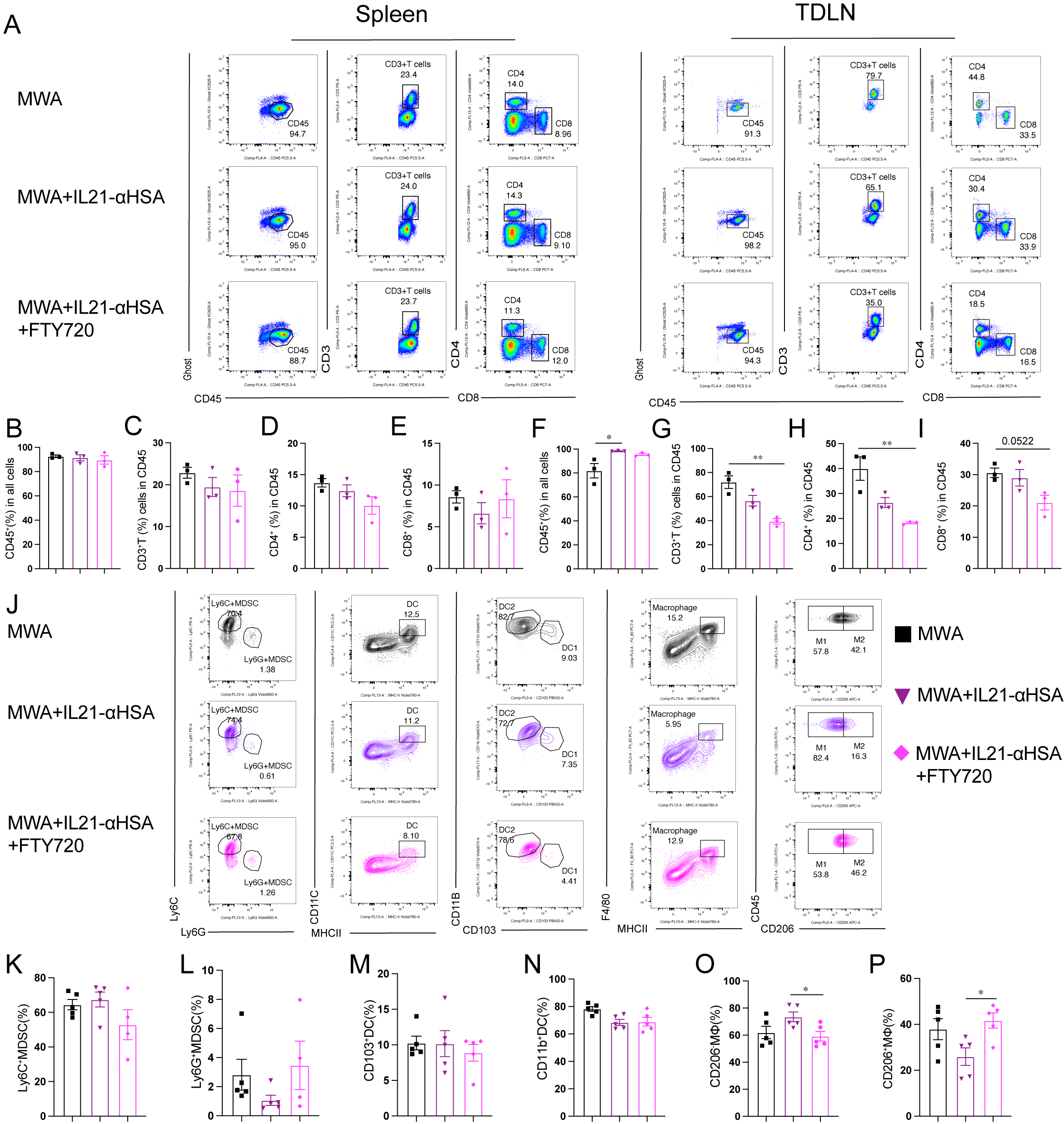

Supplement: Supplementary file 7 — Supplementary Figure 7. The enhanced anti-tumor effect of MWA combined with IL-21 requires the participation of TDLN. A–P The experimental scheme is the same as Fig. 7. A Representative flow plots of spleen and TDLN immune cell populations in different experimental groups. Quantitative percentage of spleen (B–E) and TDLN (F–I) CD45+ lymphocytes, CD3+, CD4+, and CD8+ T cells. J. Representative flow plots of tumor infiltration immune cell populations in different experimental groups. K–P Quantitative percentage of tumor infiltration MDSC, TAM1, TAM2, and DCs. (TIF 19045 KB) [file 262_2024_3718_MOESM7_ESM.tif]
